# Highly Efficient and Stable Solar Cells Based on Thiazolothiazole and Naphthobisthiadiazole Copolymers

**DOI:** 10.1038/srep14202

**Published:** 2015-09-23

**Authors:** Masahiko Saito, Itaru Osaka, Yasuhito Suzuki, Kazuo Takimiya, Takashi Okabe, Satoru Ikeda, Tsuyoshi Asano

**Affiliations:** 1 Department of Applied Chemistry, Graduate School of Engineering, Hiroshima University, 1-4-1 Kagamiyama, Higashi-Hiroshima, Hiroshima, 739-8527, Japan; 2 Emergent Molecular Function Research Group, RIKEN Center for Emergent Matter Science, Wako, Saitama 351-0198, Japan; 3 Precursory Research for Embryonic Science and Technology (PRESTO), Japan Science and Technology Agency, Chiyoda-ku 102-0075 Japan; 4 Central Technical Research Laboratory, JX Nippon Oil & Energy Corporation, 8, Chidoricho, Naka-ku, Yokohama 231-0815, Japan

## Abstract

A critical issue in polymer-based solar cells (PSCs) is to improve the power conversion efficiency (PCE) as well as the stability. Here, we describe the development of new semiconducting polymers consisting of thiophene, thiazolothiazole and naphthobisthiadiazole in the polymer backbone. The polymers had good solubility and thus solution-processability, appropriate electronic structure with narrow band gaps of ~1.57 eV and low-lying HOMO energy levels of ~−5.40 eV, and highly ordered structure with the favorable face-on backbone orientation. Solar cells based on the polymers and PC_71_BM exhibited quite high PCEs of up to 9%. More interestingly, the cells also demonstrated excellent stability as they showed negligible degradation of PCE when stored at 85˚C for 500 hours in the dark under nitrogen atmosphere. These results indicate that the newly developed polymers are promising materials for PSCs in the practical use.

Bulk heterojunction (BHJ) solar cells composed of semiconducting polymers as p-type and fullerene derivatives as n-type materials (PSCs) are of great interest as flexible and large-area renewable energy sources that can be produced by solution-processes^1^,^2^,^3^. A number of semiconducting polymers have been developed in the last decade, which have brought about significant improvement in the power conversion efficiency (PCEs)^4^,^5^,^6^,^7^,^8^. More recently, optimization of processing methods and device structures by using such polymers have led to the further improvement of PCEs reaching 10%^9^,^10^,^11^,^12^,^13^.

Important structural features desired for semiconducting polymers are well-ordered π–π stacking structure and “face-on” backbone orientation, where the backbone plane lies flat on a substrate, which facilitates the charge carrier transport along the film thickness^14^. In parallel, polymers are required to have a wide absorption range, namely a narrow band gap, to absorb as much sunlight as possible, which gives rise to the high short-circuit current density (*J*_SC_). They also need to have a low-lying highest occupied molecular orbital (HOMO) energy level to maximize the open-circuit voltage (*V*_OC_) that is proportional to the energy difference between HOMO of the semiconducting polymer and the lowest unoccupied molecular orbital (LUMO) of the fullerene derivative^15^,^16^. To fulfill these requirements, donor-acceptor (D-A) polymers consisting of an electron-rich unit (donor; D) and an electron-poor unit (acceptor; A) have been widely investigated^17^,^18^,^19^,^20^.

Recently, we have reported that a D-A copolymer system based on alkylthiophenes and thiazolothiazole (TzTz)^21^,^22^,^23^,^24^,^25^,^26^ as the D and A units, respectively (PTzBTs, Fig. 1), showed relatively high PCEs of ~7.5% in conventional cells^27^,^28^. The high performance most likely originates in the ordered and close π–π stacking structure as well as the face-on orientation. Although PTzBTs have low HOMO energy levels of ca. −5.2 eV resulting in high *V*_OC_s of ~0.9 V in solar cells, their relatively wide band gap of 1.8 eV, *i.e.*, absorption range of up to 675 nm, limited *J*_SC_s to ~13 mA cm^–2^. Thus, for the further improvement of PCE the band gap of PTzBTs must be narrowed while maintaining or even lowering the HOMO energy level. In order to realize such electronic structure, a more electron-poor unit, *i.e.*, a stronger A unit, should be introduced into the backbone.

In this work, we report on a D-A polymer system incorporating naphthobisthiadiazole (NTz)^6^,^14^,^29^,^30^,^31^,^32^ as the stronger A unit in addition to the thiophene-TzTz backbone (PTzNTzs, Fig. 1). As NTz is a rigid and π–extended fused ring, the new polymer system is expected to have a well-ordered π–π stacking structure as well as an appropriate electronic structure as mentioned above. We describe the synthesis, electronic structure, and ordering structures of PTzNTzs and their solar cell performances. We also note that, interestingly, the solar cells based on PTzNTzs showed excellent thermal stability compared to the cells based on PTzBTs.

## Results

All polymers were synthesized via the Stille coupling reaction using the stannylated TzTz monomers with the 2-ethylhexyl group (EH) or the 2-butyloctyl group (BO) on the thiophene rings as R^1^ (1a-1b) and the brominated NTz monomers with BO and the 2-hexyldecyl group (HD) on the thiophene rings as R^2^ (2a-2b) (Scheme S1). Thus, PTzNTz with four different side chain combinations were synthesized (PTzNTz-EHBO, -EHHD, -BOBO, -BOHD). The solubility was significantly different between PTzNTz-EHBO and others; whereas PTzNTz-EHBO was soluble in hot chlorobenzene (CB), or *o*-dichlorobenzene (DCB), PTzNTz-EHHD, −BOBO, and −BOHD was soluble even in chloroform (CF), CB and DCB below ca. 40 °C. Interestingly, the solubility of PTzNTzs was improved compared with PTzBTs despite the fact that NTz with a more π–extended ring than TzTz was introduced into the backbone. For example, in order to prepare a 5 g/L solution in CB, whereas PTzBT-BOHD required heating at around 100 °C, PTzNTz-BOHD, with the same side chain combination, did not require heating. The number-average molecular weight (*M*_n_), evaluated by GPC at 140, of PTzNTz-EHBO, -EHHD, -BOBO and -BOHD were about 30–50 kDa with the polydispersity index (PDI) of around 2 (Table 1). Thermal property of the polymers was studied by differential scanning calorimetry (DSC). It was revealed that whereas PTzNTz-EHHD, -BOBO and - BOHD showed transition peaks between 250–300 °C, PTzNTz-EHBO did not show transition peaks below 350 °C (Figure S6). This implies that PTzNTz-EHBO has a more rigid structure and strong aggregation nature than the others most likely due to the shorter alkyl side chains, which seemingly correlate with the solubility difference.

**Figure 2:**
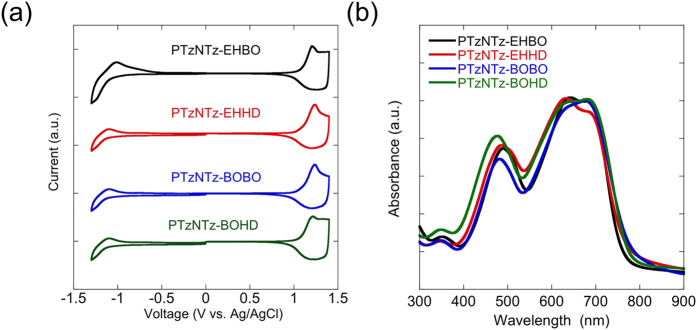
Cyclic voltammograms (a) and UV-vis absorption spectra (b) of the polymer thin films.

The HOMO energy level (*E*_HOMO_) and the LUMO energy level (*E*_LUMO_) of the polymers were evaluated by cyclic voltammetry (Fig. 2a) using the polymer thin films. All the polymers showed similar voltammograms, suggesting that the effect of the side chain is negligible. The oxidation and reduction onset potentials were about 1.07–1.08 V and −0.89–−0.91 V, respectively, which correspond to *E*_HOMO_ of −5.40–−5.41 eV and *E*_LUMO_ of −3.44–−3.46 eV (Table 1). As expected from the introduction of the strong acceptor unit NTz, *E*_HOMO_s of PTzNTzs (ca. −5.40 eV) were lower than those of PTzBTs (ca. −5.31 eV, Table S2). The downward shift of *E*_LUMO_ from PTzBTs (−3.25 eV, Table S2) to PTzNTz was more pronounced than that of *E*_HOMO_, which is natural because, in general, the acceptor unit affects more on LUMO than HOMO^33^. *E*_HOMO_ was also evaluated by photoelectron spectroscopy in air (PESA). *E*_HOMO_ was around −5.30 eV, which was lower by ca. 0.1 eV than that of PTzBTs, which is consistent with the results obtained from CV.

Figure 2b displays the UV-vis absorption spectra of the polymers in the thin film. All the polymers exhibited similar spectra with the absorption range of 400–800 nm. The absorption maximum (*λ*_max_) was ca. 680 nm (Table 1), which was red-shifted from that of PTzBTs by 60 nm. The absorption edge (*λ*_edge_) was determined to be ca. 790 nm, which corresponds to the optical band gap (*E*_g_) of 1.57–1.58 eV (Table 1). These values were about 0.2 eV smaller than that of PTzBTs. Thus, as expected from the molecular design, PTzNTzs have both the narrower *Eg* and lower *E_HOMO_* than PTzBTs.

**Figure 3:**
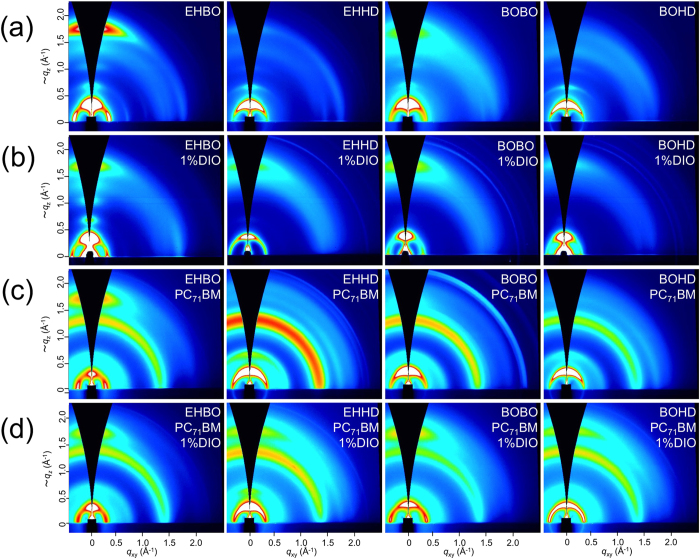
2D GIXD patterns of polymer-only films (a), DIO (1%)-aided polymer-only films (b), polymer/PC_71_BM blend films (c), and DIO (1%)-aided polymer/PC_71_BM blend films (d). The alkyl groups are shown at the right top of each image.

The ordering structure of the polymers was investigated by the X-ray diffraction studies^34^. Two-dimensional grazing incidence X-ray diffraction (2D GIXD) images of the polymer-only films and the polymer/[6,6]-phenyl-C_71_-butyric acid methyl ester (PC_71_BM) blend films on the indium tin oxide (ITO)/ZnO substrate are shown in Fig. 3. In the polymer-only films (Fig. 3a), a diffraction corresponding to the π–π stacking structure (*q* ≈ 1.65–1.70 Å^−1^) clearly appeared on the *q*_z_ axis for PTzNTz-EHBO and -BOBO, indicating that they formed crystalline domains with a favorable face-on orientation. The π–π stacking distances (*d*_π_) of these polymers were 3.69 and 3.72 Å, respectively (Figure S8, Table S3). In the meantime, the π–π stacking diffraction for PTzNTz-EHHD and -BOHD appeared very weak and *d*_π_ was wider for PTzNTz-EHHD and -BOHD with 3.77 and 3.80 Å, respectively (Figure S8, Table S3), indicating that the crystallinity was low. The difference in *d*_π_ indicates that, in this system, the use of the HD group as the side chain diminishes the intermolecular interaction. It is also noted that these *d*_π_ values are wider than that of PTzBTs (3.5 Å), even though NTz is a more π–extended fused ring and thus is expected to enhance the π–π stacking. This can be explained by the difference in the placement of the alkyl groups. In PTzBTs, all the alkyl groups on the thiophene rings point toward TzTz. In PTzNTzs, on the other hand, whereas the alkyl groups on the thiophene rings neighboring TzTz point toward TzTz, those neighboring NTz point outward from NTz. The *less* regularly placed alkyl groups as such in PTzNTzs compared to the regularly placed alkyl groups in PTzBTs could somehow weaken the π–π interaction. The difference of crystallinity between PTzNTzs and PTzBTs apparently correlates to the difference of solubility. In addition, in particular, when the alkyl groups are longer than those of PTzNTz-EHBO, the interaction becomes weaker and in turn the solubility becomes significantly higher.

In the polymer/PC_71_BM blend films fabricated from CB solutions (Fig. 3c), only PTzNTz-EHBO showed a clear π–π stacking diffraction along the *q*_z_ axis. *d*_π_ was unchanged by blending with PC_71_BM. The PTzNTz-BOBO blend film also showed a π–π stacking diffraction, but the intensity was quite weak. The results in the blend films are consistent with those in the polymer-only films. In contrast, when 1,8-diiodooctane (DIO) was used for the film fabrication as the solvent additive^35^, all the polymers exhibited a diffraction corresponding to the π–π stacking of face-on crystallites (Fig. 3d), indicating that the ordering structure was enhanced particularly for PTzNTz-EHHD, -BOBO, and -BOHD. The different behavior between PTzNTz-EHBO and the others in the DIO-aided films can be explained as follows. In the case of PTzNTz-EHHD, -BOBO, and -BOHD, with the higher solubility and thus weaker aggregation nature, the addition of DIO may slow down the crystallization of PC_71_BM, during which time the polymers crystallize as typically seen for many polymers^36^. It is also noted that the addition of DIO even enhances the crystallization of PTzNTz-EHHD, -BOBO, and -BOHD in the polymer-only films (Fig. 3b), as the π–π stacking diffraction appeared clear and the lamellar diffraction appeared as spot-like compared to the polymer-only films without DIO. In contrast, in the case of PTzNTz-EHBO, due to the strong aggregation nature and thus the lower solubility, it well crystallizes regardless of the addition of DIO (regardless of the crystallization speed of PC_71_BM). The improved ordering structures are consistent with the solar cell performances as shown below.

**Figure 4:**
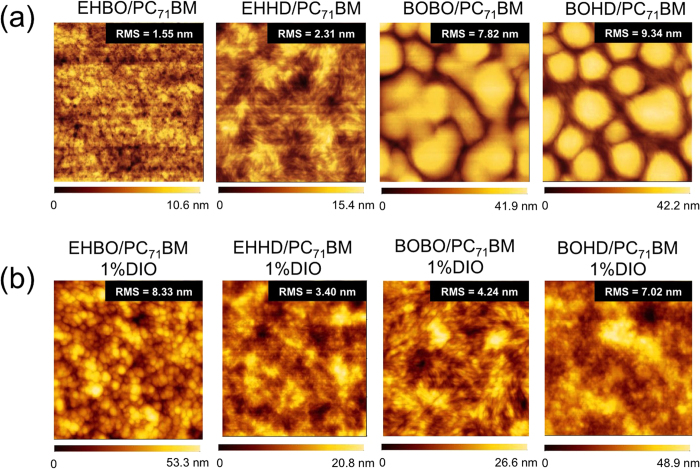
AFM images of PTzNTz/PC_71_BM blend films (**a**) and DIO (1%)-aided PTzNTz/PC_71_BM blend films (**b**).

We investigated the surface morphology of the polymer/PC_71_BM blend films by the atomic force microscopy (AFM) (Fig. 4). In the blend films fabricated with CB (Fig. 4a), whereas the PTzNTz-EHBO film formed well phase-separated morphologies, which would enlarge the polymer/PC_71_BM interface area and thus ensure the charge separation, other polymers, in particular PTzNTz-BOBO and -BOHD, formed large domains that is detrimental to the charge separation. In the DIO-aided blend films (Fig. 4b), on the other hand, whereas no morphological change was observed for PTzNTz-EHBO, drastic improvement of the morphology was observed for other polymers. These results are also in good agreement with the solar cell performances of the polymers as described below.

**Figure 5:**
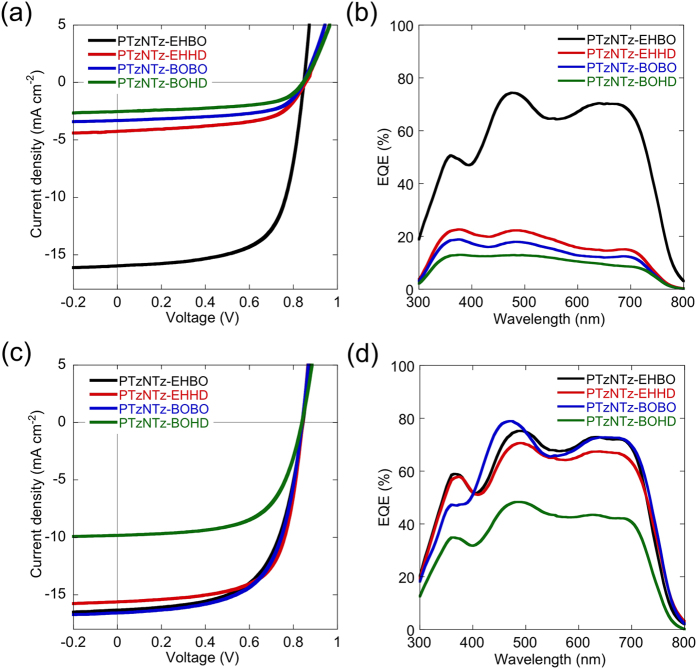
*J*–*V* curves (a,c) and EQE spectra (b,d) of the solar cells based on PTzNTzs. (**a,b**) The active layer was spun from the CB solution. (**c,d**) The active layer was spun from the CB/DIO (1 v/v%) solution.

Solar cells with an inverted structure, ITO/ZnO/active layer/MoO_x_/Ag, were used to investigate the photovoltaic properties of the polymers. PC_71_BM was used as the n-type material, and the active layer was fabricated from the CB solution. The optimal polymer to PC_71_BM weight ratio was 1:1.5 for all polymers. The current density (*J*)-voltage (*V*) curves and the external quantum efficiency (EQE) spectra of the cells under 1 sun of simulated AM 1.5G solar irradiation (100 mW/cm^2^) are displayed in Fig. 5a,b, respectively. The photovoltaic parameters are summarized in Table 2. All the cells showed similar *V*_OC_ of 0.84–0.85 V. Although *E*_HOMO_ of PTzNTzs was lower than that of PTzBT by 0.1 eV, *V*_OC_s of the PTzNTzs cells were slightly decreased by 0.03–0.04 V. The origin of the energetic loss is yet unknown. The PTzNTz-EHBO cells gave relatively high *J*_SC_ of ~16.0 mA cm^–2^ compared to that of the PTzBT cells (~13 mA cm^–2^)^28^. This is mainly due to the wider spectral response of the PTzNTz-EHBO cell covering 400–800 nm as seen in the EQE spectrum. Interestingly, the cells using PTzNTz-EHHD, -BOBO, and -BOHD exhibited significantly lower *J*_SC_ of around 2–4 mA cm^–2^, which is consistent with the EQE spectra. It is found that the fill factor (FF) was higher for the cell using PTzNTz-EHBO (0.67) than those using the other polymers (<0.6). The higher *J*_SC_ and FF of the PTzNTz-EHBO cell than the others agrees well with the results of the 2D GIXD and AFM studies, as the PTzNTz-EHBO blend film showed a higher crystalline structure and a better phase separated structure than the others when fabricated from CB solutions. As a result, PTzNTz-EHBO cells exhibited PCEs of up to 9.0% (*J*_SC_ = 16.0 mA cm^–2^, *V*_OC_ = 0.84 V, FF = 0.67), and those of the other polymer cells were 1.2–2.1%.

Note that, however, PCEs of the cells using PTzNTz-EHHD, -BOBO and -BOHD were markedly improved when the active layer was fabricated from CB solution including 1% of DIO as the solvent additive (Fig. 5c). In particular, the DIO-aided cells using PTzNTz-EHHD and -BOBO showed significant increase in *J*_SC_ to ~16.3 and ~15.6 mA cm^–2^, respectively, which were similar to the value of the cells using PTzNTz-EHBO. The increase in *J*_SC_ was consistent with the increase in EQE (Fig. 5d). FF was also increased by the addition of DIO to above 0.6 for the PTzNTz-EHHD and -BOBO cells. Thus, PCEs of the cells using these polymers were greatly improved with the association of DIO: 2.1% to 8.8% for PTzNTz-EHHD, 1.7% to 8.8% for PTzNTz-BOBO, and 1.2% to 5.2% for PTzNTz-BOHD (Table 2). PCE of the PTzNTz-EHBO-based cells fabricated with DIO was mostly similar to those without DIO. The improved performances for the cells using these polymers are in good agreement with the changes observed in the 2D GIXD and AFM studies.

**Figure 6:**
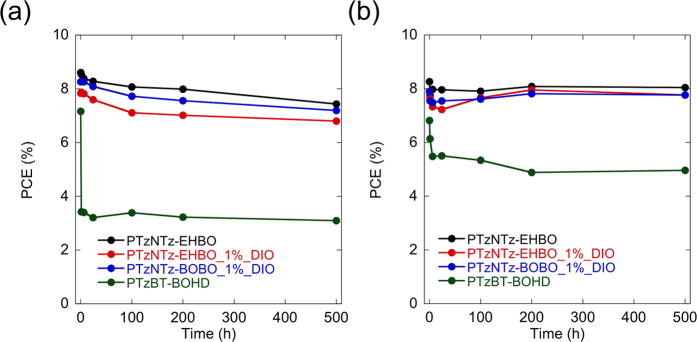
Change of PCE for the cells using PTzNTz-EHBO fabricated by CB and CB/DIO (1 v/v%), PTzNTz-BOBO fabricated by CB/DIO (1 v/v%), and PTzBT-BOHD fabricated by CB under the storage for 500 hours at 85 °C in the glovebox. MoO_x_ (**a**) and WO_x_ (**b**) were used as the hole transport layer of the cells.

We also tested the stability of the cells using PTzNTz-EHBO fabricated with and without DIO and PTzNTz-BOBO fabricated with DIO in comparison with the cells using PTzBT-BOHD. There have been established several procedures for testing device stability based on the consensus reached by the consortia of the international summit on OPV stability (ISOS)^37^,^38^. In this work, we chose to test our cells in the dark at 85 °C, which refers to category ISOS-D-3^37^. To avoid degradation concerning the encapsulation such as an adhesive material, we tested the cells without encapsulation and stored them on a hotplate in a glovebox. Figure 6a shows the change of PCE as a function of the storage time. PCE of the PTzNTz-EHBO cell decreased gradually from 8.6% to 7.5% after 500 hours, which corresponds to a 13% drop from initial PCE. The DIO-aided PTzNTz-EHBO cell showed similar behavior with a PCE drop of 13%, where PCE decreased from 8.6% to 7.5%, suggesting that DIO does not affect the device stability under these conditions for the PTzNTz system. The DIO-aided PTzNTz-BOBO cell also showed similar behavior with a PCE drop of 13%, where PCE decreased from 8.3% to 7.2%, implying that the effect of the side chain is negligible. In sharp contrast, PCE of the PTzBT-BOHD cell showed a significant drop at the initial stage; PCE decreased from 7.2% to 3.4% after 1 hour, though it showed only a slight degradation thereafter, resulting in an overall drop of 57% after 500 hours. The significant difference in the thermal stability between the PTzNTzs cells and the PTzBT-BOHD cell originates in the difference in *V*_OC_ and FF (Figure S12), although we do not yet understand the nature of the difference.

Interestingly, when the hole transport layer, MoO_x_, was replaced with WO_x_, the thermal stability of the cells were greatly improved (Fig. 6b). All the PTzNTzs cells tested here with the initial PCE of 8.3%, 7.9%, and 7.9% for PTzNTz-EHBO, PTzNTz-EHBO with DIO, and PTzNTz-BOBO with DIO, respectively, showed negligible degradation after 500 hours. The drop of PCE for the PTzBT-BOHD cell was 30% after 500 hours (PCE decreased from 6.8% to 4.8%). The main factor of the stability improvement for the PTzNTzs cells was the improved *J*_SC_ drop (Figure S12). The improvement in the PTzBT-BOHD cell was mainly due to the improved *V*_OC_ drop, though *J*_SC_ was also slightly improved (Figure S12). The difference between the cells with MoO_x_ and WO_x_ is currently under investigation. Overall, regardless of the hole transport layer, the PTzNTzs cells are much more stable than the PTzBT-BOHD cell. To date there have been some reports showing the stability of PSCs under various conditions^39^,^40^,^41^,^42^. To the best of our knowledge, this is the first report for PSCs to demonstrate such high PCEs of ~9% and almost perfect stability under a standardized conditions at the same time. These results suggest that the introduction of NTz into the polymer backbone is advantageous for the thermal stability of the solar cells as well as PCE.

## Conclusion

New thiophene-thiazolothiazole-naphthobisthiadiazole semiconducting polymers (PTzNTzs) were synthesized and characterized, and their photovoltaic characteristics were investigated. The solubility of PTzNTzs were greatly improved from that of PTzBTs. PTzNTzs had both narrow band gaps (ca. 1.57 eV) and low-lying HOMO levels (ca. −5.40 eV) compared to thiophene-thiazolothiazole polymers (PTzBTs) that have been reported before. PTzNTz-EHBO with the shortest side chain combination exhibited the best PCE of as high as 9.0% in the inverted solar cells among the polymers synthesized here. Although the cells based on PTzNTz-BOBO and -EHHD with the longer side chains exhibited very low PCEs of ~2%, the use of DIO at the time of fabrication as the solvent additive greatly improved their PCEs to ~8.8%. The difference of the solar cell behavior between these polymers with different side chain combination is likely attributed to the difference in crystallinity. Interestingly, the PTzNTzs cells demonstrated excellent stability as they showed negligible degradation after 500 hour-storage at 85 °C under N_2_ atmosphere. To the best of our knowledge, the PTzNTzs cells are the best performing PSCs in terms of having high PCE and high stability at the same time. These results indicate that PTzNTzs are promising polymers for practical application.

## Methods

### Materials

2,5-Bis(3-(2-ethylhexyl-5-(trimethylstannyl)thiophen-2-yl)thiazolo[5,4-*d*]thiazole (1a)^28^ and 2,5-bis(3-(2-butyloctyl-5-(trimethylstannyl)thiophen-2-yl)thiazolo[5,4-*d*]thiazole (1b)^28^ 5,10-bis(5-bromo-4-(2-butyloctyl)thiophen-2-yl)naphtho[1,2-*c*:5,6-*c*´]bis[1,2,5]thiadiazole (2a)^31^ and 5,10-bis(5-bromo-4-(2-hexyldecyl)thiophen-2-yl)naphtho[1,2-c:5,6-c´]bis[1,2,5]thiadiazole (2b)^31^ were synthesized according to the reported procedure. Pd(PPh_3_)_4_ (PPh_3_ = triphenylphosphine) was purchased from Tokyo Chemical Industry, Co., Ltd. (TCI), and used as purchased. Toluene was purified by a Glass Contour Solvent System prior to use. Polymerization was carried out with a microwave reactor, Biotage Initiator. Molecular weight of the polymers was evaluated by a high-temperature GPC (140 °C), TOSOH HLC-8121GPC/HT, using DCB as the eluent and polystyrene standard.

### Synthesis of the polymers

To a reaction tube equipped with a stirring bar, the stannylated TzTz monomer (0.10 mmol), the dibrominated NTz monomer (0.10 mmol), Pd(PPh_3_)_4_ (2.3 mg, 0.002 mmol), and CB (5 mL) were added. Then the tube was purged with argon and sealed. The reaction tube was set into a microwave reactor and heated to 180 °C for 40 min. After cooling to room temperature, the reaction solution was poured into 200 mL of methanol containing 5 mL of hydrochloric acid, and stirred for 5 hours. Then the precipitated solid was subjected to the sequential Soxhlet extraction with methanol and hexane, to remove low molecular weight fractions. The residue was then extracted with chloroform for PTzNTz-EHHD, - BOBO, and - BOHD, and CB for PTzNTz-EHBO, and reprecipitated in 200 mL of methanol to yield dark green solid (yield = 68% for PTzNTz-EHBO, 89% for -EHHD, 86% for -BOBO, 75% for -BOHD). Anal. Calcd for C_70_H_92_N_6_S_8_ (PTzNTz-EHBO): C, 65.99; H, 7.28; N, 6.60. Found: C, 65.65; H, 7.15; N, 6.31. Calcd for C_78_H_108_N_6_S_8_ (-EHHD): C, 67.58; H, 7.85; N, 6.06. Found: C, 67.64; H, 7.80; N, 5.93. Calcd for C_78_H_108_N_6_S_8_ (-BOBO): C, 67.58; H, 7.85; N, 6.06. Found: C, 67.62; H, 7.74; N, 5.98. Calcd for C_86_H_124_N_6_S_8_ (-BOHD): C, 68.93; H, 8.34; N, 5.61. Found: C, 68.55; H, 8.20; N, 5.45.

### Solar Cell Fabrication and Measurement

Patterned ITO substrates (purchased from Atsugi Micro) were first pre-cleaned sequentially by sonicating in a detergent bath, de-ionized water, acetone, and isopropanol at rt, and in boiled isopropanol each for 10 min, and then baked at 120 °C for 10 min in air. The substrates were then subjected to a UV/ozone treatment at rt for 20 min. ZnO layer was prepared by spin-coating (at 5000 rpm, 30 sec) a precursor solution prepared from zinc acetate dehydrate (0.5 g) and ethanolamine (0.14 mL) in 5 mL of 2-methoxyethanol. ZnO substrates were immediately baked at 200 °C for 30 min in air, and then rinsed with acetone, isopropanol and boiled isopropanol for 10 min. The photoactive layer was deposited in a glove box (KOREA KIYON, KK-011AS-EXTRA) by spin coating. CB solution containing 5~7 g/L of the polymer sample with respective amount of PC_71_BM was kept at 100 °C for 30 min. The hot CB solution was directly spin-coated on the substrate at 600 rpm for 20 sec. The thin films were transferred into a vacuum evaporator (ALS Technology, E-100J) connected to the glove box, and the MoO_x_ layer (7.5 nm) or WO_x_ layer (15 nm) was deposited, followed by the deposition of the Ag layer (100 nm). The active area of the cells was 0.16 cm^2^. *J*–*V* characteristics of the cells were measured using a Keithley 2400 source-measure unit in nitrogen atmosphere under 1 sun (AM1.5 G) conditions using a solar simulator (SAN-EI Electric, XES-40S1). The light intensity was calibrated with a reference PV cell (KONICA MINOLTA AK-100 certified at National Institute of Advanced Industrial Science and Technology, Japan). EQE spectra were measured with a Spectral Response Measuring System (SOMA OPTICS, S-9241). More than 10 different devices were made and measured to collect the photovoltaic properties. For thermal stability tests, cells were stored on a hotplate at 85 °C in the glovebox. *J*–*V* characteristics were measured after 1, 2, 6, 24, 100, 200 and 500 hours.

## Additional Information

**How to cite this article**: Saito, M. *et al.* Highly Efficient and Stable Solar Cells Based on Thiazolothiazole and Naphthobisthiadiazole Copolymers. *Sci. Rep.* **5**, 14202; doi: 10.1038/srep14202 (2015).

## Supplementary Material

Supplementary Information

## Figures and Tables

**Figure 1:**
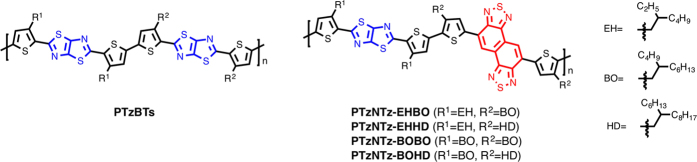
Chemical structure of semiconducting polymers based on thiophene and thiazolothiazole (PTzBTs), and thiophene, thiazolothiazole and naphthobisthiadiazole (PTzNTzs).

**Table 1:** Polymerization results^a^ and electronic properties of the polymers.

Polymer	*M*_n_(kDa)	*M*_w_(kDa)	PDI	*E*_HOMO_ (eV)		*E*_LUMO_(eV)^b^	*λ*_max_ (nm)	*λ*_edge_ (nm)/*E*_g_^opt^(eV)^d^
				CV^b^	PESA^c^			
PTzNTz-EHBO	32.7	64.3	2.0	−5.41	−5.28	−3.45	490, 643, 677	784/1.58
PTzNTz-EHHD	47.0	91.8	2.0	−5.40	−5.28	−3.46	487, 633, 675	787/1.58
PTzNTz-BOBO	51.6	113.2	2.2	−5.40	−5.30	−3.46	482, 678	791/1.57
PTzNTz-BOHD	29.1	59.2	2.2	−5.41	−5.29	−3.44	477, 680,	788/1.57

^*a*^Determined by high temperature GPC (DCB, 140 °C) using polystyrene standard.

^*b*^HOMO and LUMO energy levels determined by cyclic voltammetry.

^*c*^HOMO energy levels evaluated by photoelectron spectroscopy in air (PESA).

^*d*^*λ*_edge_: absorption edge, *E*_g_: optical band gap.

**Table 2:** Photovoltaic properties of the solar cells based on PTzNTzs/PC_71_BM.

Polymer	DIO	*J*_SC_(mA cm^−2^)	*V*_OC_(V)	FF	PCE_max_[PCE_ave_](%)^a^
PTzNTz-EHBO	–	16.0	0.84	0.67	9.0 [8.7]
	1%	16.3	0.84	0.62	8.5 [8.1]
PTzNTz-EHHD	–	4.3	0.85	0.58	2.1 [1.9]
	1%	15.6	0.84	0.67	8.8 [8.5]
PTzNTz-BOBO	–	3.3	0.85	0.60	1.7 [1.5]
	1%	16.6	0.84	0.63	8.8 [8.5]
PTzNTz-BOHD	–	2.5	0.84	0.58	1.2 [1.1]
	1%	9.8	0.84	0.63	5.2 [4.8]

^*a*^PCE_max_: maximum power conversion efficiencies, PCE_ave_: average power conversion efficiencies.
