# Supplementary material for: Highly Efficient and Stable Solar Cells Based on Thiazolothiazole and Naphthobisthiadiazole Copolymers
Source: Sci Rep. 2015 Sep 23;5:14202. doi: 10.1038/srep14202 (PMC4585800; doi:10.1038/srep14202)
Supplement: Supplementary Information [file srep14202-s1.doc]

**Supporting Information**

Highly Efficient and Stable Solar Cells Based on Thiazolothiazole and Naphthobisthiadiazole Copolymers

Masahiko Saito^†^, Itaru Osaka,*^,‡^, ^§^ Yasuhito Suzuki,^‡^ Kazuo Takimiya,^†, ‡^ Takashi Okabe,^⊥^

Satoru Ikeda,^⊥^ Tsuyoshi Asano^⊥^

^†^*Department of Applied Chemistry, Graduate School of Engineering, Hiroshima University,*

*1-4-1 Kagamiyama, Higashi-Hiroshima, Hiroshima, 739-8527, Japan*

^‡^*Emergent Molecular Function Research Group, RIKEN Center for Emergent Matter Science, Wako, Saitama 351-0198, Japan*

^§^*Precursory Research for Embryonic Science and Technology (PRESTO), Japan Science and Technology Agency, Chiyoda-ku 102-0075. Japan*

^⊥^*Central Technical Research Laboratory, JX Nippon Oil & Energy, 8, Chidoricho, Naka-ku, Yokohama 231-0815, Japan*

*E-mail address: [itaru.osaka@riken.jp](mailto:itaru.osaka@riken.jp)

**Instrumentation.**

NMR spectra were measured in deuterated *o*-dichlorobenzene with a JNM-ECS-400 spectrometer. Cyclic voltammetry (CV) was carried out with an ALS Electrochemical Analyzer Model 612D in acetonitrile containing tetrabutylammonium hexafluorophosphate (Bu_4_NPF_6_, 0.1 M) as supporting electrolyte at a scan rate of 100 mV/s. Counter and working electrodes were made of Pt, and the reference electrode was Ag/AgCl. All potentials were calibrated with the standard ferrocene/ferrocenium redox couple (Fc/Fc^+^:E^1/2^ = +0.47 V measured under identical condition). HOMO energy levels *(E*_HOMO_) and LUMO energy levels *(E*_LUMO_) were calculated with the following equations:

*E*_HOMO_ (eV) = -4.33 - *E*_ox_

*E*_LUMO_ (eV) = -4.33 + *E*_red_

where *E*_ox_ and *E*_red_ are the onset oxidation and reduction potential of cyclic voltammograms, respectively. *E*_HOMO_ was also determined from the onset of photoelectron spectra measured by a photoelectron spectrometer, model AC-2, in air (Riken Keiki Co., Ltd). UV-vis absorption spectra were measured using a Shimadzu UV-3600 spectrometer. Thermal analyses were carried out with differential scanning calorimetry (DSC) on an EXSTAR DSC7020 (SII Nanotechnology, Inc.) at 10 °C/min for both heating and cooling processes. Dynamic force-mode atomic force microscopy study was carried out on a Nanocute scanning probe microscope system (SII Nanotechnology, Inc.). 2D GIXD experiments were conducted at the SPring-8 on the beamline BL46XU. The sample was irradiated at a fixed incident angle on the order of 0.12° through a Huber diffractometer with the X-ray energy of 12.39 keV (λ = 1 Å). 2D GIXD patterns were recorded with a 2D image detector (Pilatus 300K). Samples for the X-ray measurements were prepared by spin-casting the polymer and polymer/PC_71_BM solution on the ITO/ZnO substrate with the same conditions as the solar cell fabrication.

**OFET Fabrication and Measurement.**

All film fabrication processes except substrate cleaning were performed in a glovebox. Heavily doped n^+^ -Si (100) wafers with 200 nm-thick thermally grown SiO_2_ (*C*_i_ = 17.3 nF cm^-2^ ) were used for the substrate. The Si/SiO_2_ substrates were ultrasonicated with acetone and isopropanol for 20 min. The cleaned substrates were treated with 1*H*, 1*H*, 2*H*, 2*H*-perfluorodecyltriethoxysilane (FDTS) to form a self-assembled monolayer, in which the wafers were exposed to FDTS vapor in a closed desiccator and rinsed with toluene. The polymer layer was then spin-coated from a hot (~140 °C) 2 g/L CB solution at 1000 rpm for 10 s and then 2500 rpm for 35 s, and subsequently annealed at 150 °C for 30 min, respectively, in glove box. On top of the polymer thin films, Au drain and source electrodes (thickness 80 nm) were deposited in a vacuum through a shadow mask, where the drain-source channel length (*L*) and width (*W*) are 40 μm and 3.0 mm, respectively.

Current-voltage characteristics of the OFET devices were measured at room temperature in air with a Keithly 4200-SCS semiconductor characterization system. Field-effect mobilities were calculated in the sauration regime (drain voltage (*V*_D_) = -60 V) of the *I*_D_ using the following equation,

*I*_D_ = (W*C*_i_/2L)μ(*V*_G_ - *V*_T_)^2^

where *C*_i_ is the capacitance of the dielectric layer, *I*_D_ is source-drain current, and *V*_G_, and *V*_T_ are the source-drain gate, and threshold voltages, respectively. Current on/off ratios (*I*_on_/*I*_off_) were determined from the minimum current around *V*_G_ = 0-20V (*I*_off_) and the current at *V*_G_ = -60V (*I*_on_). The mobility data were collected from more than 10 different devices.

**Scheme S1.** Polymerization of PTzNTzs

**
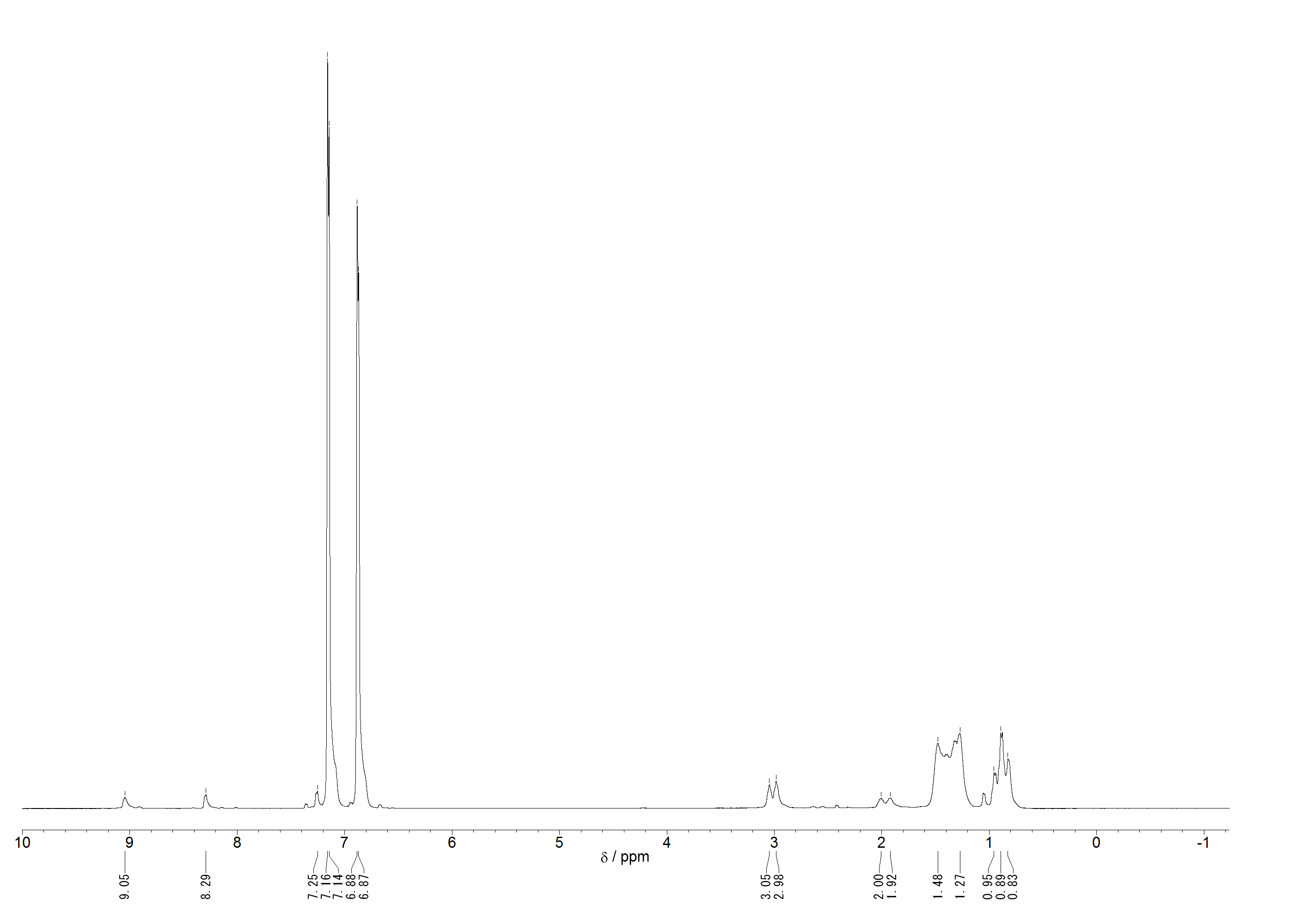
**

**Figure S1.** ^1^H-NMR spectrum of PTzNTz-EHBO


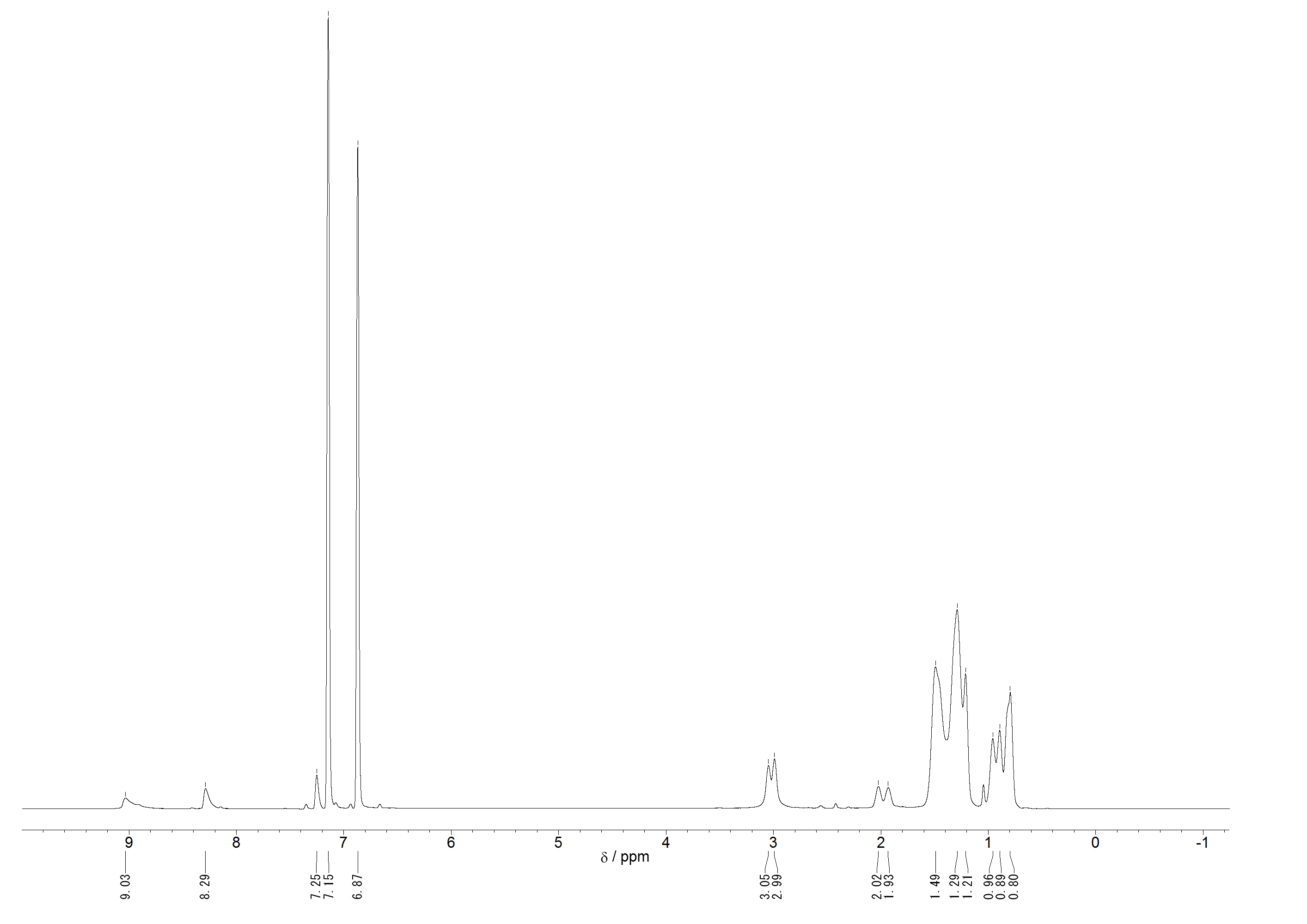


**Figure S2.** ^1^H-NMR spectrum of PTzNTz-EHHD


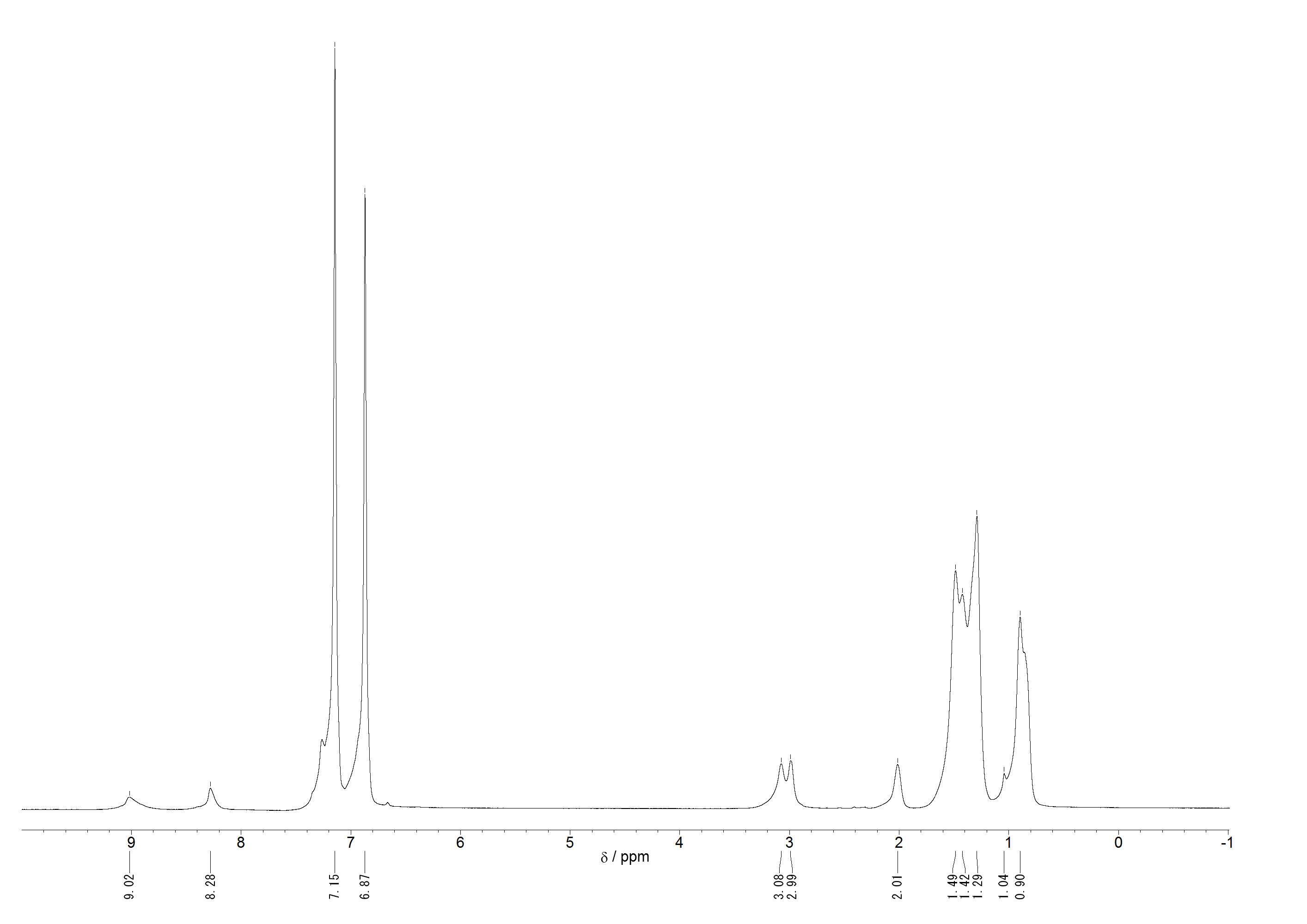


**Figure S3.** ^1^H-NMR spectrum of PTzNTz-BOBO


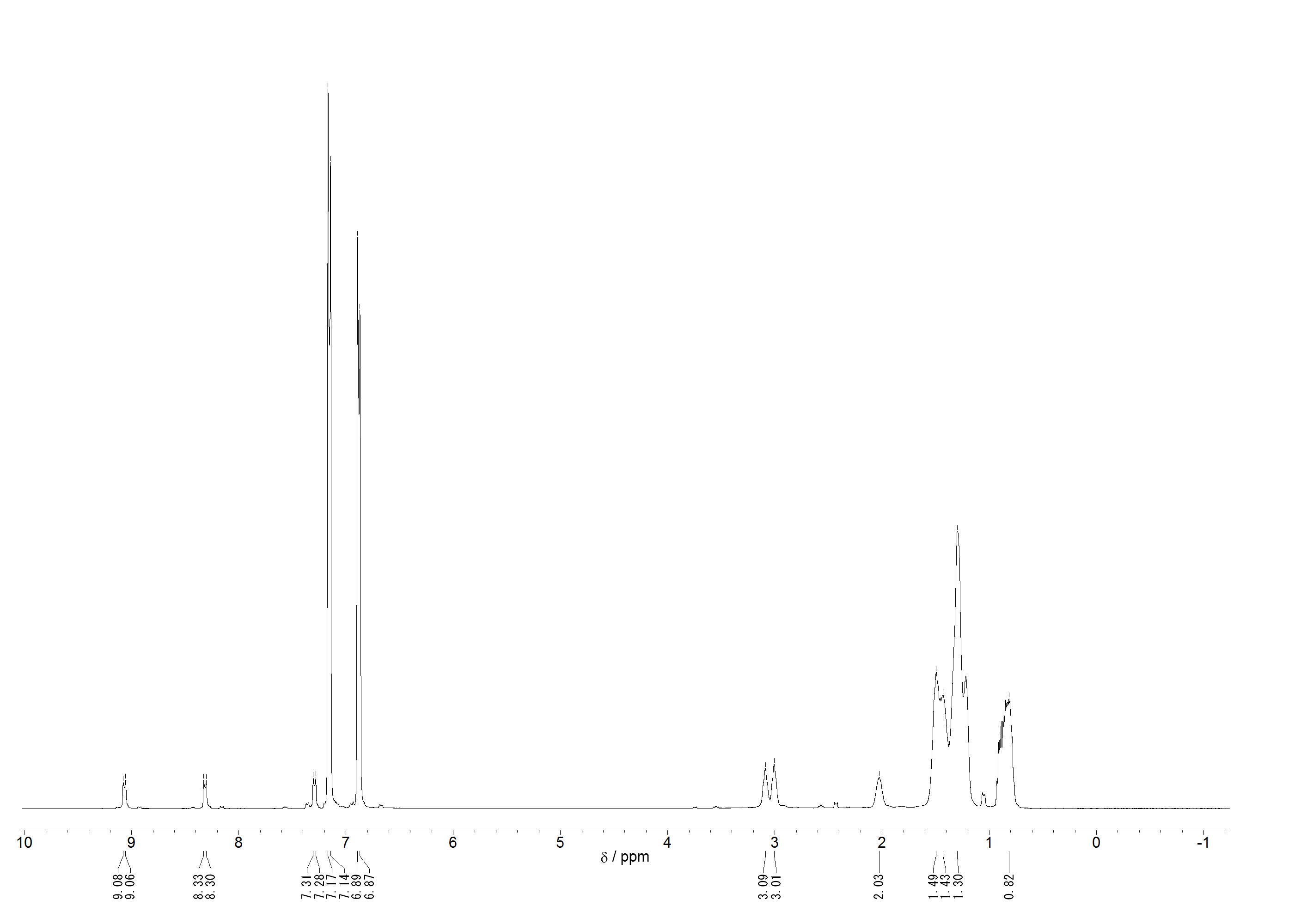


**Figure S4.** ^1^H-NMR spectrum of PTzNTz-BOHD

**Figure S5.** UV-vis absorption spectra of PTzNTz-EHBO, -EHHD, -BOBO and -BOHD in the chlorobenzene solution.

**Table S1.** Electronic properties obtained from the UV-vis spectra of the polymer solutions.

|  | *λ*_max_ (nm) | *λ*_edge_ (nm) / *E*_g_^opt^ (eV) *^a^* |
| --- | --- | --- |
| PTzNTz-EHBO | 495, 644 | 764 / 1.62 |
| PTzNTz-EHHD | 489, 653 | 767 / 1.62 |
| PTzNTz-BOBO | 485, 666 | 772 / 1.61 |
| PTzNTz-BOHD | 484, 660 | 771 / 1.61 |


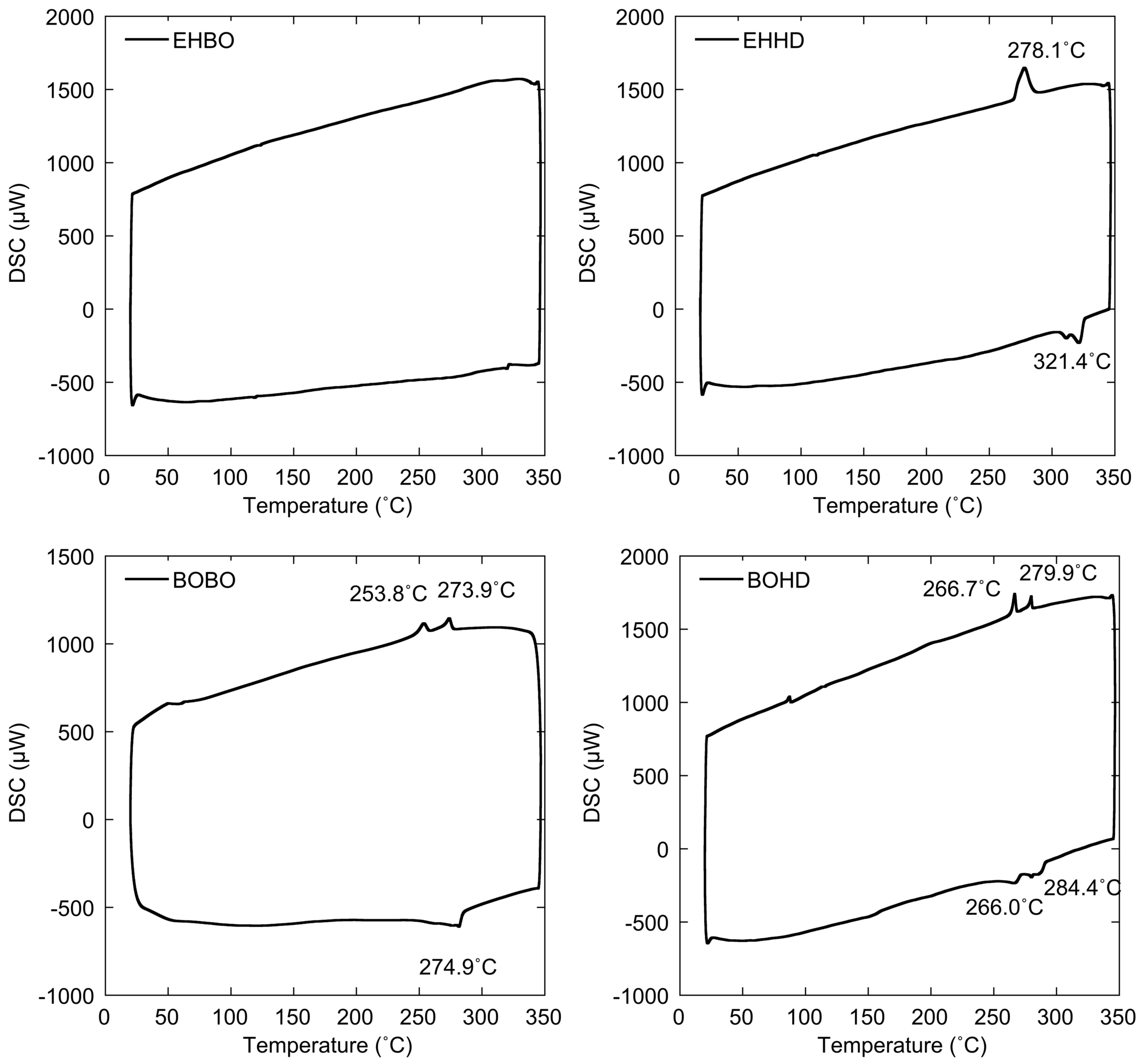


**Figure S6.** DSC thermograms of polymers PTzNTz-EHBO, EHHD, BOBO and BOHD at a temperature ramp rate of 10 °C min^-1^


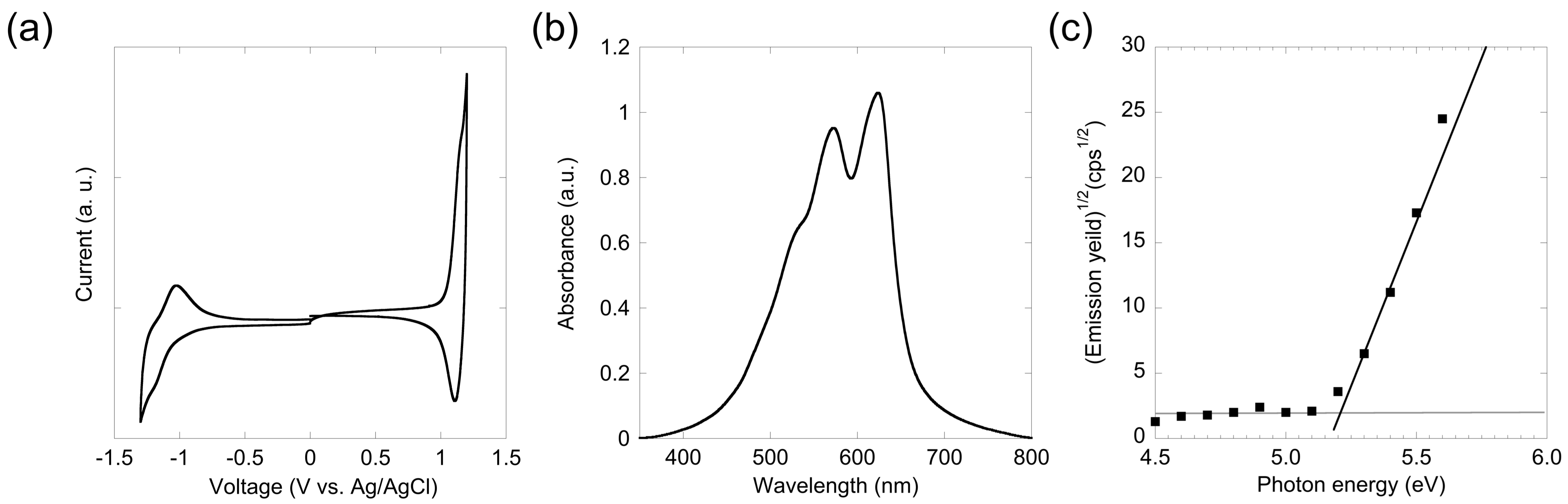


**Figure S7.** Cyclic voltammogram (a), UV-vis absorption spectrum (b) and photoelectron spectra (c) of a PTzBT-BOHD thin film.

***Table S2.*** *Electronic properties of PTzBT-BOHD*

| *E*_HOMO_ (eV)*^a^* | *E*_LUMO_ (eV)*^a^* | *λ*_max_ (nm) | *λ*_edge_ (nm) / *E*_g_^opt^ (eV) *^b^* | *E*_HOMO_ (eV) *^c^* |
| --- | --- | --- | --- | --- |
| -5.31 | -3.25 | 573, 624 | 673 / 1.84 | -5.23 |

*^a^*HOMO and LUMO energy levels were determined by cyclic voltammetry. *^b^λ*_edge_: absorption edge, *E*_g_ : optical band gap. *^c^*HOMO energy levels evaluated by photoelectron spectroscopy in air (PESA).


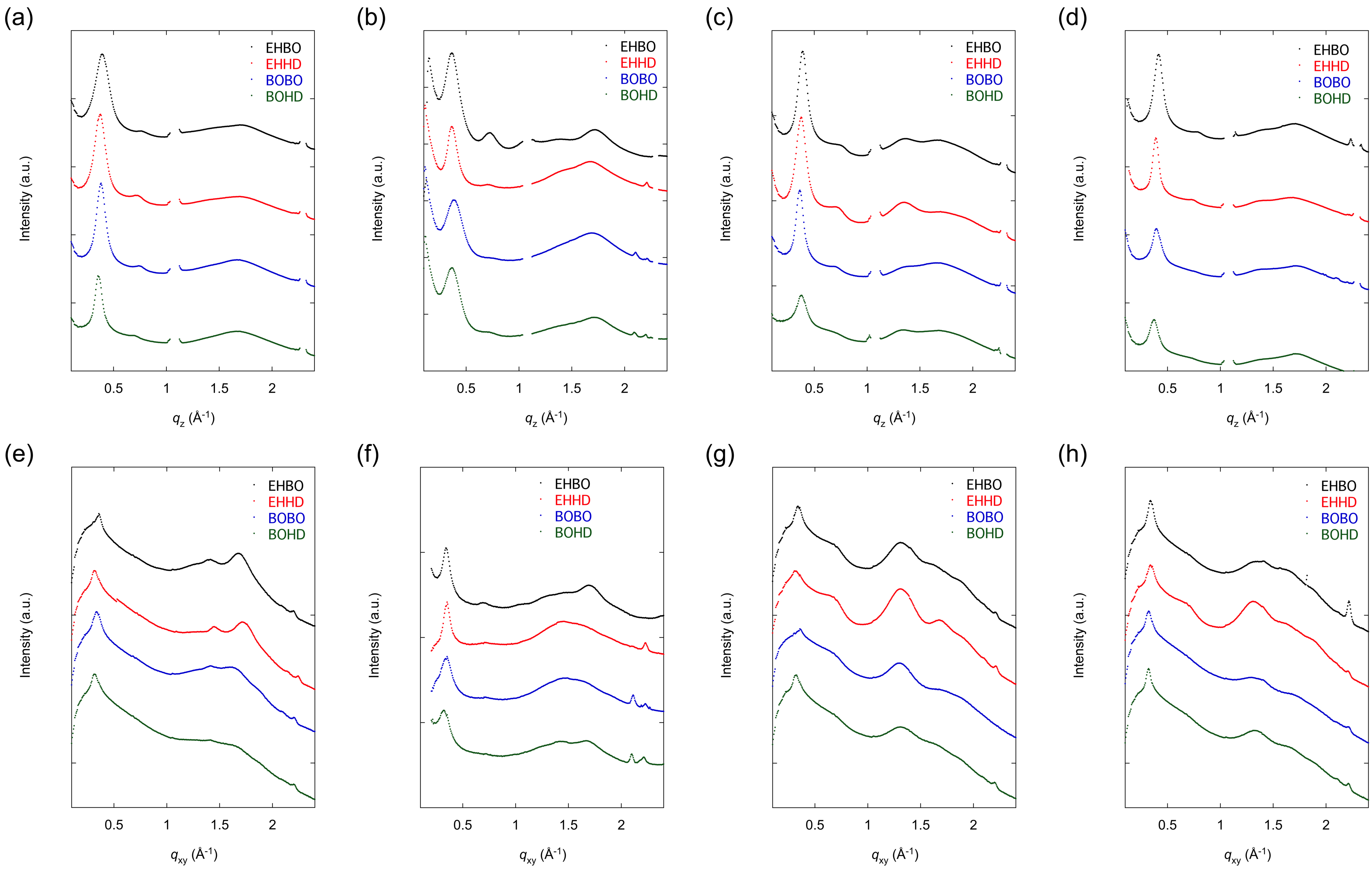


**Figure S8.** Cross-sectional profiles of the 2D GIXD patterns cut along the *q*_z_ axis for polymer-only films (a), DIO-(1%)-aided polymer-only films (b), polymer/PC_71_BM blend films (c), and DIO-(1%)-aided polymer/PC_71_BM blend films (d). Cross-sectional profiles of the 2D GIXD patterns cut along the *q*_xy_ axis for polymer-only film (e), DIO-(1%)-aided polymer-only films (f), polymer/PC_71_BM blend films (g), and DIO-(1%)-aided polymer/PC_71_BM blend films (h).

**Table S3.** *d*-Spacing of the lamellar structure (*d*_l_) and π–π stacking (*d*_π_), and full width at half maxima (FWHM) evaluated from GIXD profiles for PTzNTzs.

| Polymer | *d*_lamella_ (Å)^[a]^ | *d*_π_ (Å)^[b]^ | FWHM^[c]^ |
| --- | --- | --- | --- |
| -EHBO | 16.9 | 3.69 | 0.042 |
| -EHHD | 18.5 | 3.77 | 0.087 |
| -BOBO | 17.5 | 3.72 | 0.059 |
| -BOHD | 18.6 | 3.80 | 0.063 |
| -EHBO (1% DIO) | 17.0 | 3.69 | 0.031 |
| -EHHD (1% DIO) | 18.5 | 3.77 | 0.061 |
| -BOBO (1% DIO) | 17.7 | 3.71 | 0.055 |
| -BOHD (1% DIO) | 18.7 | 3.80 | 0.061 |
| -EHBO/PC_71_BM | 17.1 | 3.69 | 0.040 |
| -EHHD/PC_71_BM | 18.6 | 3.78 | 0.097 |
| -BOBO/PC_71_BM | 18.0 | 3.73 | 0.089 |
| -BOHD/PC_71_BM | 19.0 | 3.81 | 0.089 |
| -EHBO/PC_71_BM (1% DIO) | 17.0 | 3.69 | 0.045 |
| -EHHD/PC_71_BM (1% DIO) | 18.6 | 3.78 | 0.091 |
| -BOBO/PC_71_BM (1% DIO) | 17.7 | 3.73 | 0.083 |
| -BOHD/PC_71_BM (1% DIO) | 18.9 | 3.81 | 0.091 |

[a] Lamella distance (*d*_lamella_) was determined from the diffraction profile along the *q*_xy_ axis (in-plane) of the 2D GIXDs images. [b] π–π stacking distance (*d*_π_) was determined from the diffraction profile along the *q*_z_ axis (out-of-plane) of the 2D GIXDs images. [c] Full-width half-maximum (FWHM) are determined from the lamellar of the diffraction *q*_xy_ axis (in-plane).


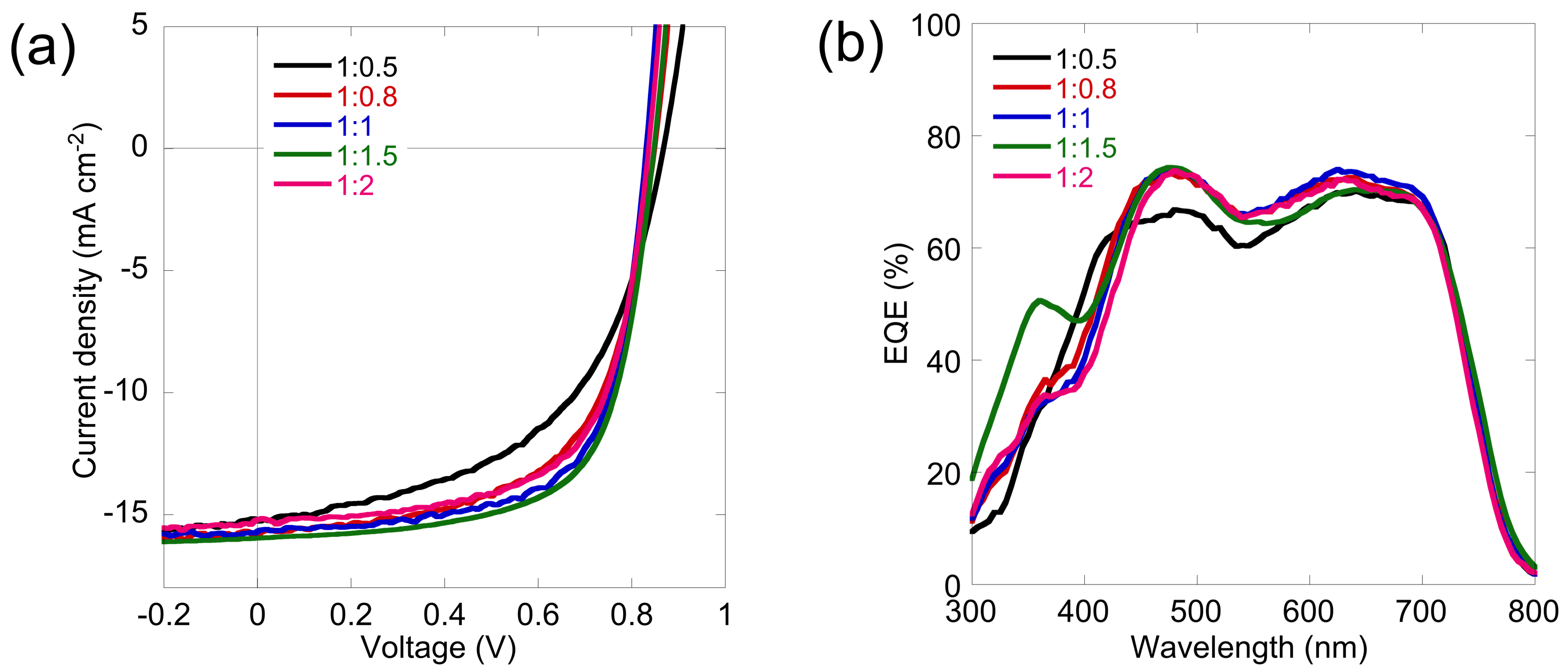


**Figure S9.** *J*–*V* curves (a) and EQE spectra (b) of the solar cells based on PTzNTz-EHBO with different polymer to PC_71_BM (p:n) ratio.

**Table S4.** Photovoltaic properties of the solar cells based on PTzNTz-EHBO.

| p:n ratio | *J*_SC_ (mA cm^–2^) | *V*_OC_ (V) | FF | PCE_max_ [PCE_ave_] (%)*^a^* |
| --- | --- | --- | --- | --- |
| 1:0.5 | 15.26 | 0.87 | 0.53 | 7.0 [6.8] |
| 1:0.8 | 15.81 | 0.85 | 0.61 | 8.2 [8.0] |
| 1:1 | 15.69 | 0.83 | 0.67 | 8.8 [8.6] |
| 1:1.5 | 15.97 | 0.84 | 0.67 | 9.0 [8.7] |
| 1:2 | 15.21 | 0.83 | 0.66 | 8.4 [8.1] |

*^a^* PCE_max_: maximum power conversion efficiencies, PCE_ave_: average power conversion efficiencies.


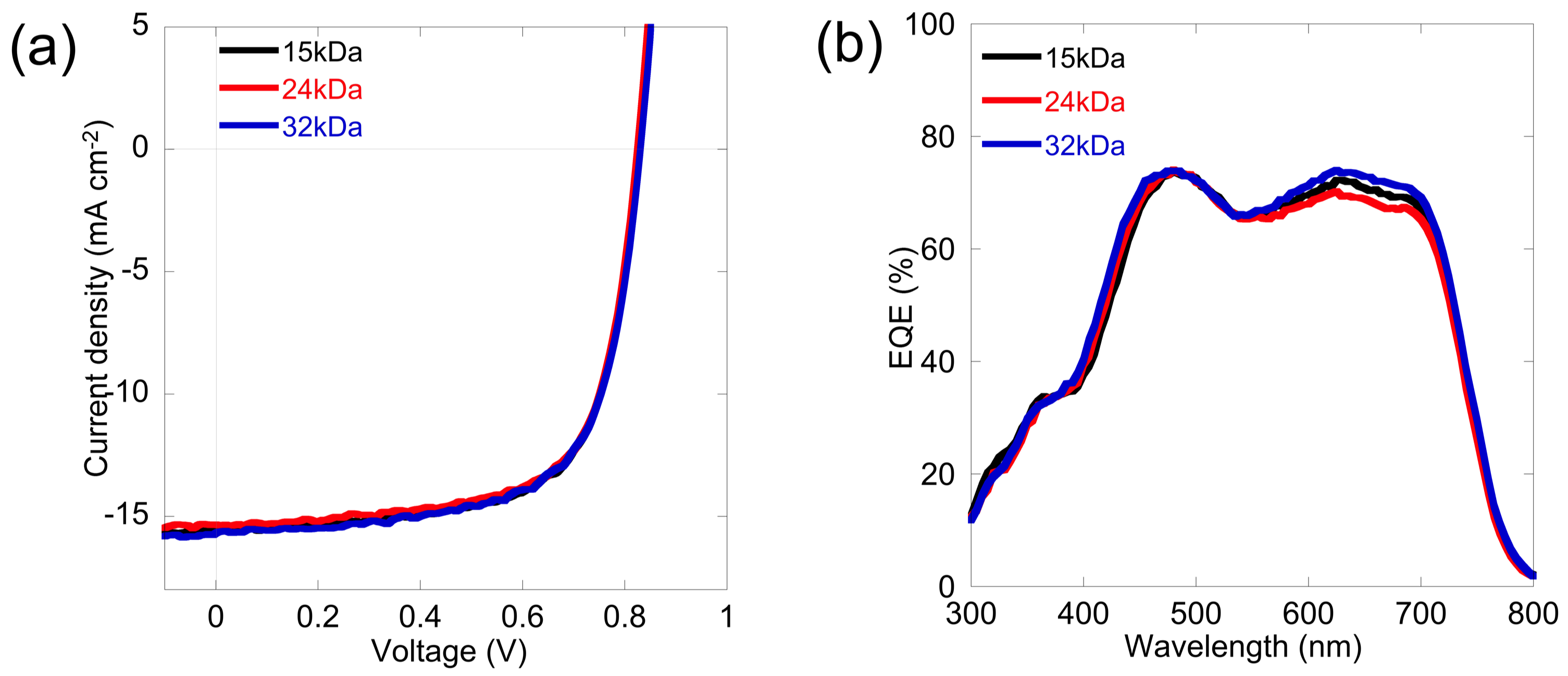


**Figure S10.** *J*−*V* curves (a) and EQE spectra (b) of the solar cells based on PTzNTz-EHBO with different molecular weight.

**Table S5.** Photovoltaic properties of the solar cells based on PTzNTz-EHBO.

| *M*_n_ (kDa) | *J*_SC_ (mA cm^–2^) | *V*_OC_ (V) | FF | PCE_max_ [PCE_ave_] (%)*^a^* |
| --- | --- | --- | --- | --- |
| 15.1 | 15.56 | 0.83 | 0.69 | 8.8 [8.5] |
| 24.4 | 15.34 | 0.83 | 0.69 | 8.7 [8.4] |
| 32.7 | 15.69 | 0.83 | 0.67 | 8.8 [8.6] |

*^a^* PCE_max_: maximum power conversion efficiencies, PCE_ave_: average power conversion efficiencies.


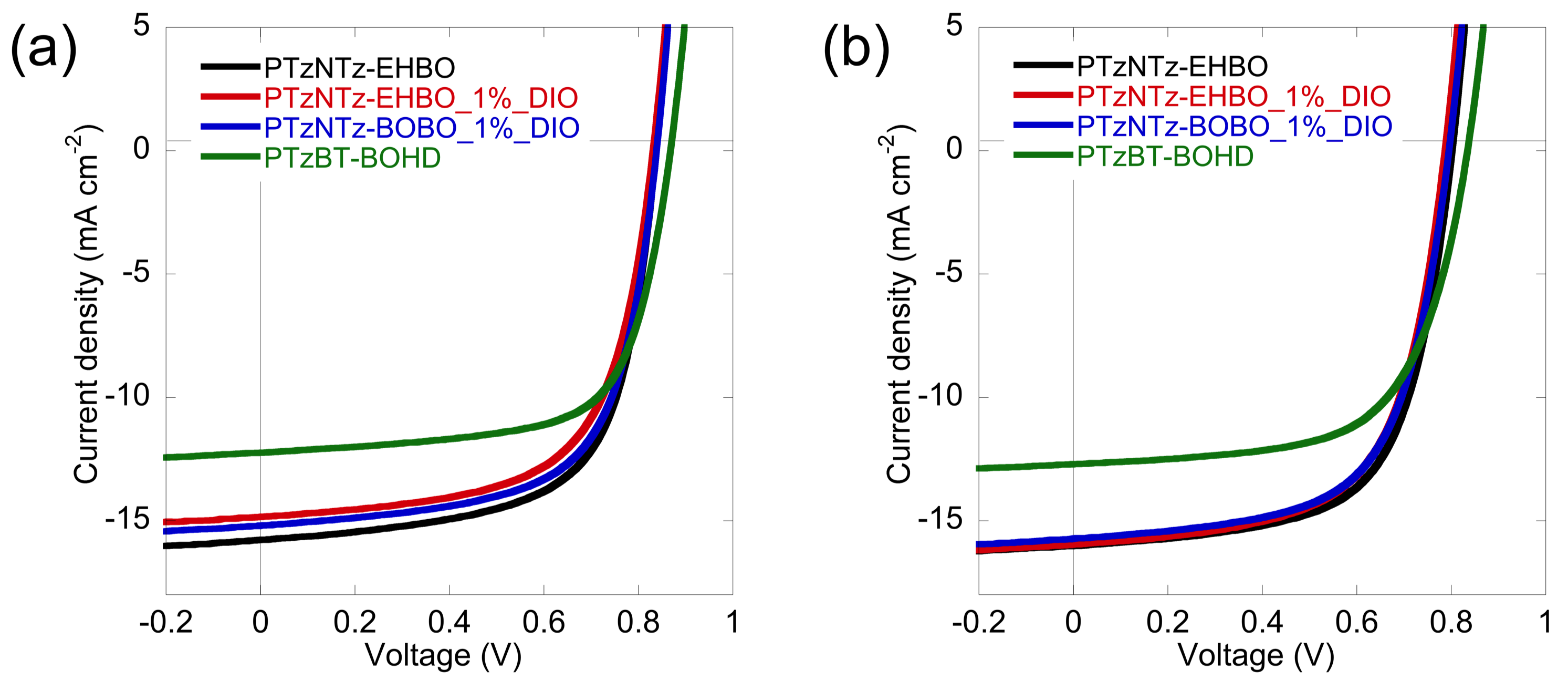


**Figure S11.** *J*−*V* curves of the solar cells using MoO_x_ (a) and WO_x_ (b) as the hole transport layer.

**Table S6.** Photovoltaic properties of the solar cells with different hole transport layer (HTL).

|  | HTL | *J*_SC_ (mA cm^–2^) | *V*_OC_ (V) | FF | PCE_max_ [PCE_ave_] (%)*^a^* |
| --- | --- | --- | --- | --- | --- |
| PTzNTz-EHBO | MoO_x_ | 15.8 | 0.84 | 0.65 | 8.6 [8.3] |
|  | WO_x_ | 16.0 | 0.80 | 0.64 | 8.3 [8.0] |
| PTzNTz-EHBO  (1% DIO) | MoO_x_ | 14.8 | 0.83 | 0.64 | 7.8 [7.5] |
|  | WO_x_ | 15.9 | 0.79 | 0.63 | 7.9 [7.7] |
| PTzNTz-BOBO  (1% DIO) | MoO_x_ | 15.2 | 0.84 | 0.65 | 8.3 [8.0] |
|  | WO_x_ | 15.8 | 0.78 | 0.63 | 7.9 [7.5] |
| PTzBT-BOHD  (1% DIO) | MoO_x_ | 12.2 | 0.87 | 0.67 | 7.2 [6.9] |
|  | WO_x_ | 12.7 | 0.84 | 0.64 | 6.8 [6.6] |

*^a^* PCE_max_: maximum power conversion efficiencies, PCE_ave_: average power conversion efficiencies.


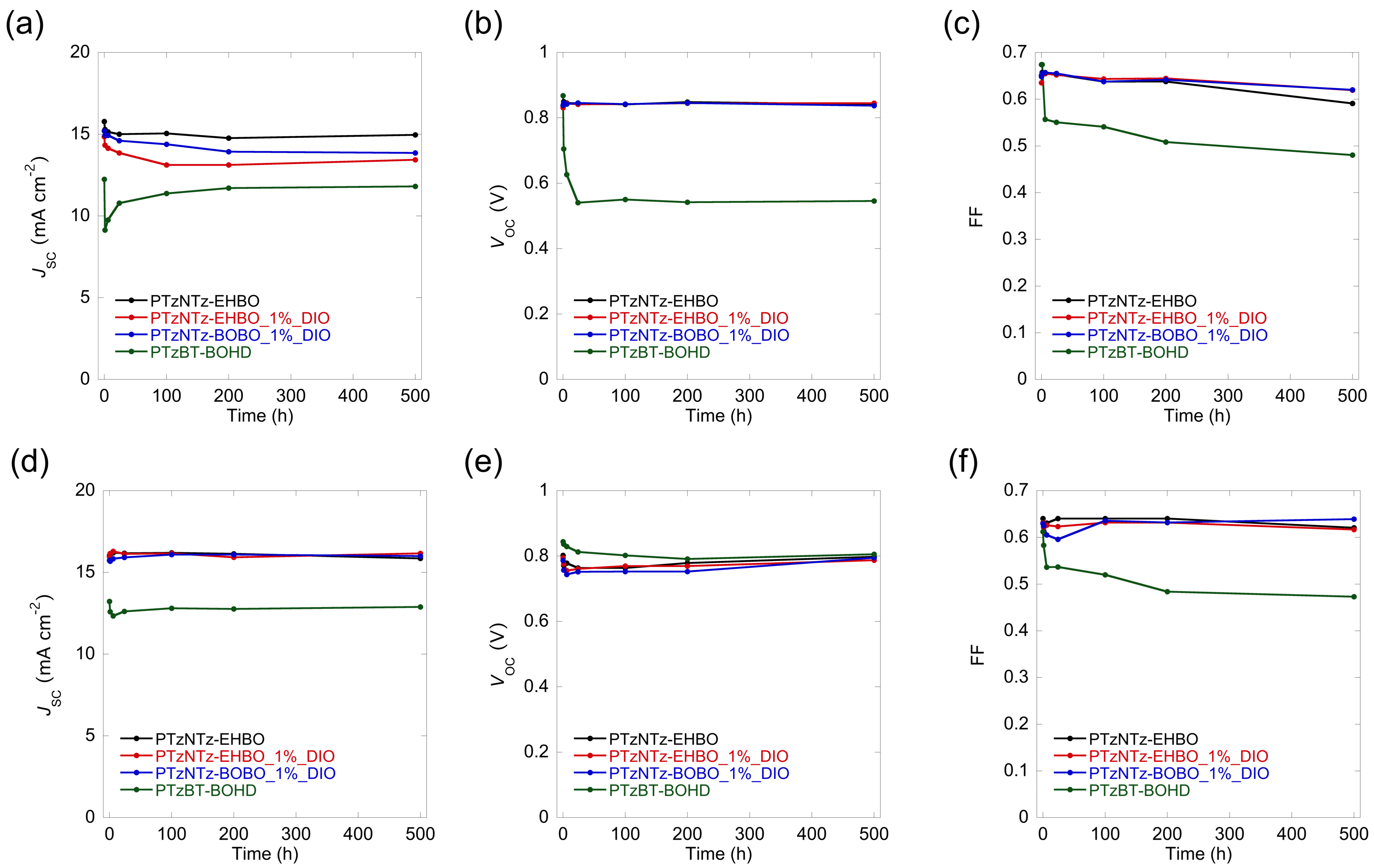


**Figure S12.** Change of *J*_SC_ (a, d), *V*_OC_ (b, e), and FF (c, f) for the cells using PTzNTz-EHBO fabricated by CB and CB/DIO (1 v/v%), PTzNTz-BOBO fabricated by CB/DIO (1 v/v%), and PTzBT-BOHD fabricated by CB under the storage for 500 hours at 85 °C in the glovebox. MoO_x_ (a–c) and WO_x_ (d–f) were used as the hole transport layer of the cells.


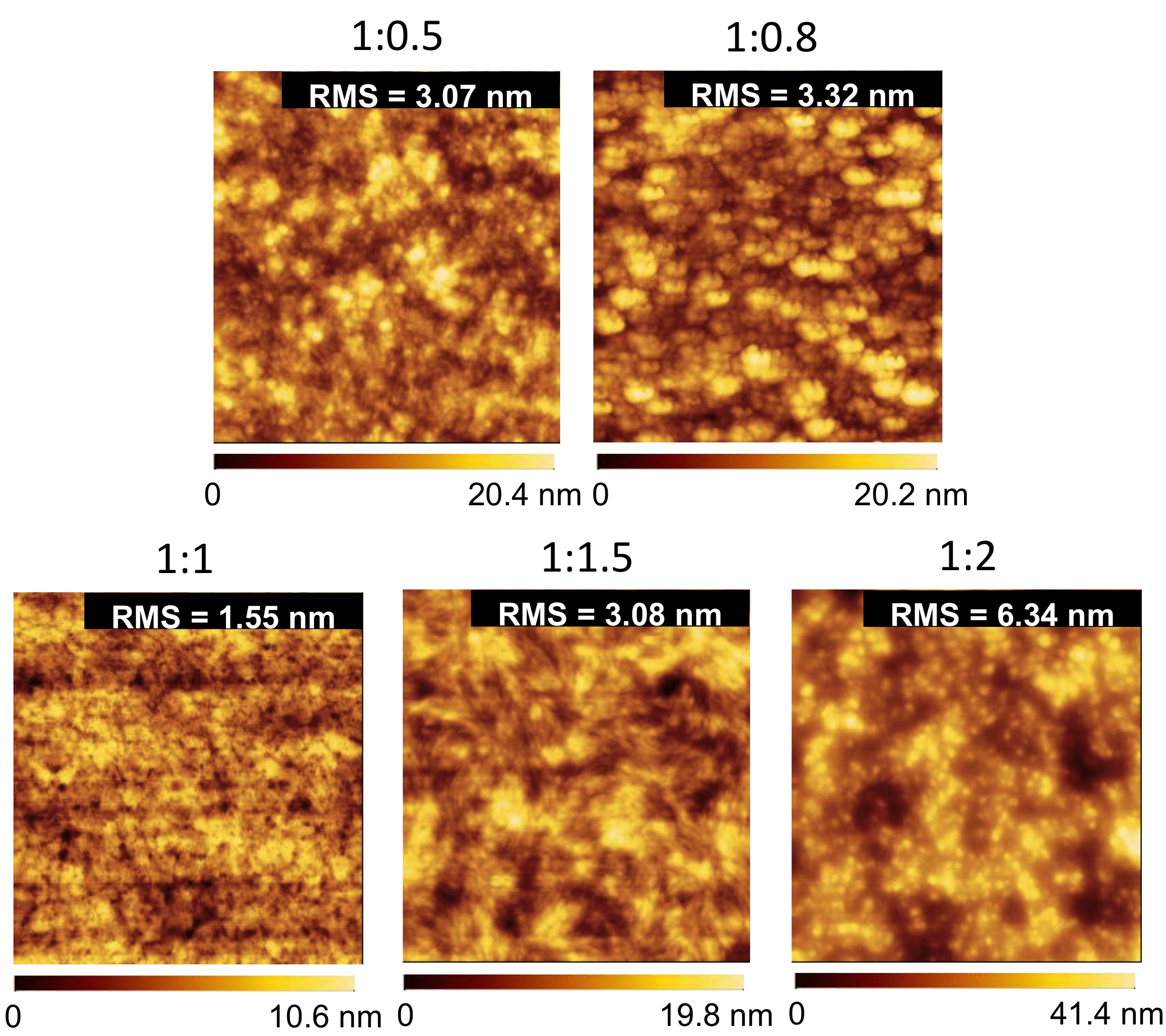


**Figure S13.** AFM image of PTzNTz-EHBO:PC_71_BM blend films with the different p:n ratio.


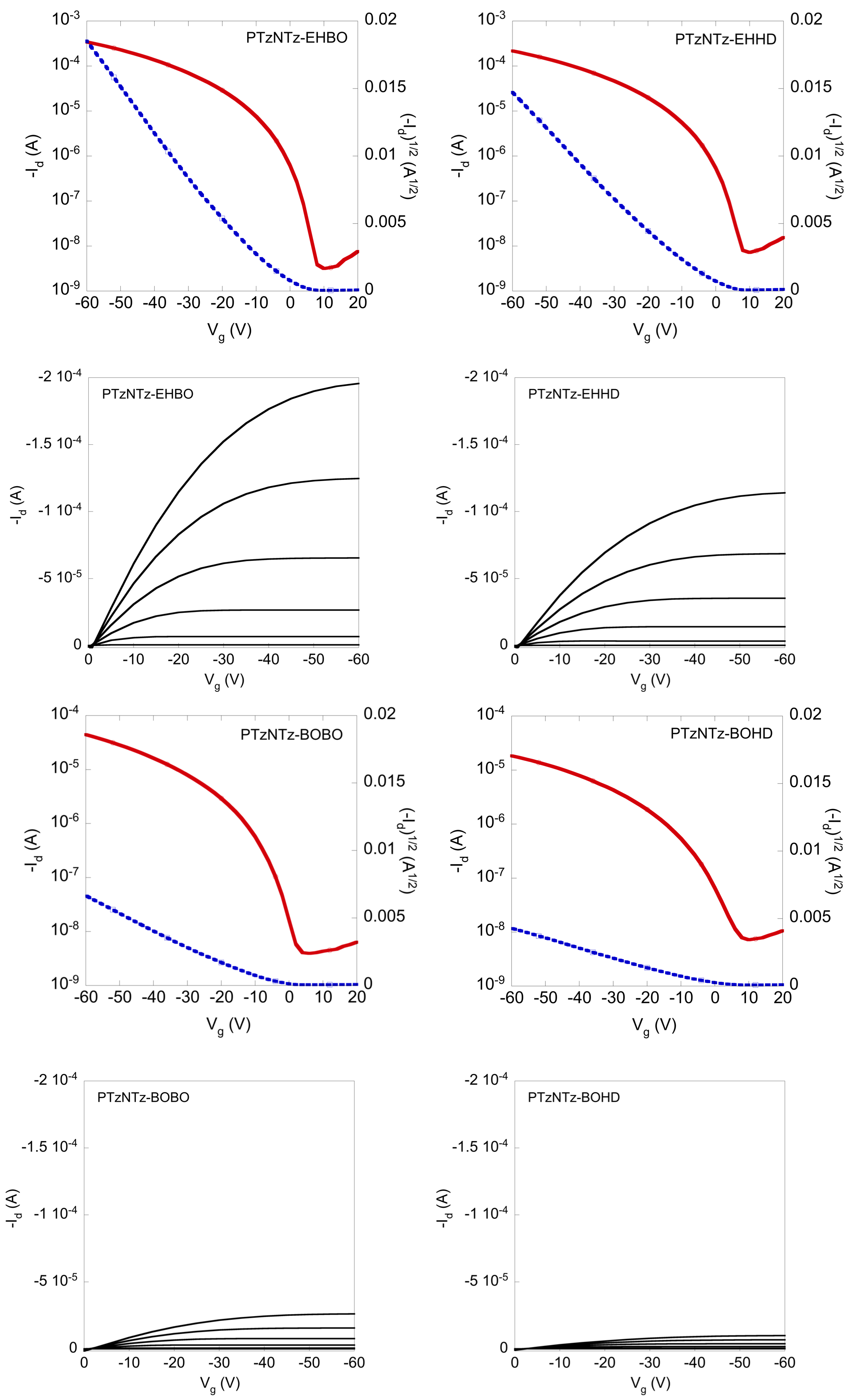


**Figure S14.** Transfer (a) and output (b) characteristics of OFET devices using PTzNTzs.

**Table S7.** Transistor properties of PTzNTzs

| Polymer | *μ* (cm^2^ V^–1^ s^–1^)^a^ | *V*_th_ (V)^b^ | *I*_on_ /*I*_off_^c^ |
| --- | --- | --- | --- |
| PTzNTz-EHBO | 0.16 | -7.2 to -0.9 | ~10^5^ |
| PTzNTz-EHHD | 0.10 | -6.4 to 0.7 | ~10^4^ |
| PTzNTz-BOBO | 0.024 | -4.0 to 0.6 | ~10^4^ |
| PTzNTz-BOHD | 0.0084 | -3.1 to 4.4 | ~10^3^ |

(a)Field-effect mobilities in the FDTS-treated device. (b) Evaluated from the onset of the transfer curves. (c)Caluclated from *I*_SD_ = -60 V (on) and 20 V (off).
